# Skill trade‐offs promote persistent individual differences and specialized tactics

**DOI:** 10.1002/ece3.10578

**Published:** 2023-10-04

**Authors:** Frédérique Dubois

**Affiliations:** ^1^ Département de Sciences Biologiques Université de Montréal Montreal Quebec Canada

**Keywords:** alternative tactics, behavioural specialization, competitiveness, individual variation, producer‐scrounger game, skill trade‐offs

## Abstract

Individuals generally differ in their ability to perform challenging behaviours, but the causes of such variability remain incompletely understood. Because animals can usually use different behavioural tactics to achieve their goals, we might expect individual differences in skill to be maintained when the available tactics require different abilities to perform well. To explore this idea, I used the producer‐scrounger (PS) paradigm, which considers interactions between foragers that may either invest effort in searching for resources (i.e. produce) or exploit others' discoveries (i.e. scrounge). Specifically, I tested whether individual differences in cognitive traits (i.e. the ability to find food) might result from a trade‐off with competitiveness (i.e. the ability to steal food) that would exert disruptive selection pressure and, as such, might explain the coexistence of condition‐dependent foraging tactics. If individuals differ in their competitiveness, with strong contestants being better able to monopolize food resources (and hence to scrounge), the model predicts that strong and weak competitors should rely more on scrounging and producing, respectively, especially when the finder's advantage is low. These findings indicate that the existence of individual differences in competitive abilities may be sufficient to explain short‐term individual foraging tactic specialization. Yet, the degree of behavioural specialization is expected to depend on both the social and ecological context. Furthermore, persistent phenotypic differences, that are necessary for stable individual specialization, require the existence of a trade‐off between competitive abilities that enable greater success as scroungers and cognitive abilities that are associated with better efficiency to detect and/or capture prey and, as such, enable greater success as producers. Therefore, this study further highlights the importance of considering the existence of alternative tactics to measure and predict the evolution of traits, including cognitive traits, within populations.

## Graphical abstract

When animals can use alternative tactics to achieve their goal, they tend to consistently differ in the degree to which they play each tactic. Using the producer‐scrounger as a general framework, here I demonstrate that the maintenance of persistent phenotypic differences, that are necessary for stable individual specialization, requires the existence of a trade‐off between abilities that enable greater success as either a producer or a scrounger.

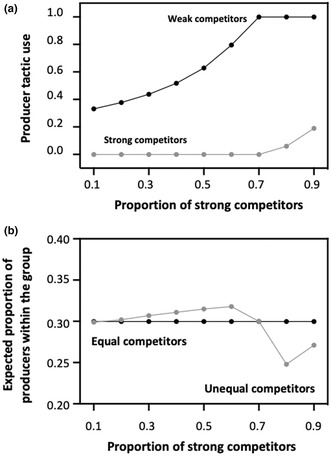

## 1 INTRODUCTION

Individuals of the same population, and thus experiencing similar ecological and social conditions, generally differ in their ability to perform challenging behaviours. For instance, individuals may differ in their capacity to perform courtship display, agonistic behaviour or cognitive tasks (e.g. Boogert et al., 2018; Lucon‐Xiccato & Bisazza, 2017; Rowe & Healy, 2014). Because performance measures can be linked to fitness, the behavioural or cognitive traits associated with greater performance are likely to evolve by natural selection. For instance, the ecological intelligence hypothesis (EIH) argues that complex foraging tactics would have evolved to deal with changes in the environment (Rosati, 2017; Sol, 2009). For that reason, one would expect species that perform better on cognitive tasks (and presumably have larger brain sizes) to occupy more unpredictable habitats where they can cope with novel or altered environmental conditions, compared to species that experience predictable environmental conditions. Evidence supporting this prediction mainly comes from comparative studies that have demonstrated that species or populations that are more dependent on caching exhibit more accurate spatial memory and enlarged hippocampal volume (Garamszegi & Eens, 2004; Provosudov & Clayton, 2002). Yet, if the capacity of individuals to perform challenging behaviour represents an adaptation to local conditions, there should be much less inter‐individual variability than observed in skills and behavioural tactic use. One reason to explain the maintenance of such variability could be that individuals, to achieve the same goal, use alternative frequency‐dependent behavioural tactics that require conflicting abilities. I tested this idea using the producer‐scrounger (PS) paradigm, which considers interactions between foragers that may either invest effort in searching for resources (i.e. produce) or exploit others' discoveries (i.e. scrounge), with the payoffs to the scrounging tactic being negative frequency dependent (Giraldeau & Caraco, 2000; Vickery et al., 1991). While it is generally assumed that an individual's phenotype has no influence on its decision or ability to play either tactic (but see Barta & Giraldeau, 1998; Philips et al., 2018), here I considered that individuals may differ in their ability to produce and scrounge, and I explored whether a trade‐off between these two skills promotes individual behavioural specialization and favours the maintenance of persistent differences in individual performances.

The PS game is frequently used to analyse strategic interactions among individuals that compete for divisible resources. As such, it assumes that a producer obtains a share of the resource it can exploit exclusively before the scroungers join (i.e. the finder's advantage). The payoff to producing, thus, increases as the finder's advantage increases, but decreases as the number of scroungers that compete for the remaining resources increases (Giraldeau & Caraco, 2000; Vickery et al., 1991). The Producer Scrounger (PS) game model predicts that social foragers should adjust their use of producing and scrounging according to ecological conditions (e.g. group size, resource value) to maximize their food intake. Assuming that group members are identical, the model predicts that the two tactics, at equilibrium, should reap equal payoffs and no player could benefit by changing its strategy *unilaterally* (i.e. while the other players keep theirs unchanged). At the group level, experimental evidence has proven the predictions of the PS game to be qualitatively correct (Afshar & Giraldeau, 2014; Giraldeau & Dubois, 2008). However, individuals, within a group, consistently differ in the degree to which they play each tactic (Aplin & Morand‐Ferron, 2017; Harten et al., 2018; Jolles et al., 2013; Morand‐Ferron et al., 2011), thus indicating that the benefits of producing or scrounging may depend on individuals' phenotype. For instance, when scrounging involves kleptoparasitism or aggressive displacement, dominant or large individuals have been found to preferentially adopt the scrounging role (Bicca‐Marques & Garber, 2005; Jones et al., 2017; Lendvai et al., 2006; Liker & Barta, 2002; Philips et al., 2018). On the other hand, when locating or capturing food is cognitively demanding, one might expect better problem‐solvers or individuals with better spatial memory to preferentially adopt the producing role (Katsnelson et al., 2011; Reichert et al., 2021). Two phenotypic‐limited PS games have previously been developed to address the question of whether differences among individuals in their competitive ability or success in stealing food may affect their use of producing and scrounging (Barta & Giraldeau, 1998; Philips et al., 2018) but without trying to understand what mechanisms contribute to the maintenance of these differences. If the benefits of producing and scrounging differ among individuals according to their ability to discover and capture food or to their fighting ability, however, they might receive different payoffs, causing a reduction in variability over time.

Up to now, consistent individual tactic specialization has mostly been established using short‐term experiments in stable groups and without directly measuring the payoffs to producer and scrounger. However, if differences among individuals in their preference for one or the other tactic are associated with innate individual differences in their fighting ability, producing might result in lower fitness than scrounging (Barta & Giraldeau, 1998; Liker & Barta, 2002). For instance, as reported by a number of experimental studies, clumped resources that can be monopolized are likely to increase agonistic interactions among group members (Grant & Guha, 1993; Monaghan & Metcalfe, 1985; Ryer & Olla, 1995) and, as such, should favour individuals with greater fighting ability that will specialize on scrounging, and whose proportion in the group will therefore increase over time. Thus, this process would tend to reduce inter‐individual differences in fighting ability unless maybe there is a trade‐off between the ability to discover food resources and the ability to aggressively defend them; the more individuals would be able to monopolize food resources, the less they would be able to discover new food resources. As a consequence, this trade‐off might favour the coexistence of weak competitors that are very efficient in prey capture and hence would specialize on producing, with strong competitors that would specialize on scrounging. A number of empirical studies have demonstrated the existence of trade‐offs between different abilities, which can affect an individual's efficiency at finding or stealing food, for example between cognitive ability and competitiveness (Kawecki, 2010; Laland & Reader, 1999). Though, it still remains unknown whether and if so, under which ecological conditions, this mechanism contributes to maintaining inter‐individual variability in individuals' skills and to generating consistent individual tactic specialization. Here, I developed a PS game model to analyse the evolution over time of the proportion of weak and strong competitors, weak competitors having in return greater chances to locate and capture prey, and their respective producer and scrounger tactic use. Specifically, I consider a group with two phenotypes that are initially present in the same proportion and differ in terms of their ability to aggressively compete for food and capacity to locate and capture food. I assume that individuals, at each time step, are able to adjust the frequency at which they use the two foraging tactics to maximize their gain according to their own phenotype and the frequency at which both tactics are used in the group. I can then estimate the average payoff of both phenotypes, deduce their proportion at the next time, and repeat the same procedure until the group reaches stable equilibria. I tested whether the amount of variation in individual skills and the degree of tactic specialization at equilibrium depend on ecological conditions (e.g. group size, energetic value of prey) as well as on the magnitude of differences among individuals.

## 2 MATERIALS AND METHODS

### 2.1 Model description

The group contains a fixed number of *G* foragers that can be of two phenotypes (i.e. strong or weak), whose initial respective proportions are $p_{0}$ and ($1-p_{0}$). Strong and weak competitors differ in terms of their ability to compete for food and their capacity to detect and capture prey (the list of parameters and phenotypic variables are presented in Tables 1 and 2). Specifically, individuals are characterized by their competitive weight, which is equal to 1 and ω (with ω≤1) for strong and weak contestants respectively. The competitive weight of an individual determines the proportion of a resource that it can monopolize when the resource is contested by at least one other group member. This proportion is then equal to $\frac{1}{1+W}$ and $\frac{\omega }{\omega +W}$ for strong and weak competitors, respectively, where *W* is the sum of the competitive weights of the other contestants. Conversely, the probability of detecting prey is set to 1 for weak competitors, while strong competitors are able to detect and capture prey with a probability δ (with δ≤1).

**TABLE 1:** List of the parameters used in the model.

Parameter	Meaning	Default value	Range of tested values
*F*	Energetic value of prey	10	5–40
a	Finder's advantage	2 (i.e. 20% of the value of prey)	0–10
G	Group size	10	5–50

**TABLE 2:** List of phenotypic variables.

Variable	Strong competitors	Weak competitors	Default value	Range of tested values
Frequency at time *t*	$p_{t}$	$1-p_{t}$	$p_{0}=0.5$	
Competitive weight	1	ω	ω=0.8	0–1
Efficiency to detect prey	δ	1	δ=0.8	0–1
Frequency of *Producer* tactic use	x	y		

The two phenotypes can adjust their use of the Producer and Scrounger tactics to maximize their gain. Thus, the parameters *x* (with 0≤x≤1) and $y\;\left(\text{with}\;0\leq y\leq 1\right)$ represent, respectively, the frequency at which strong and weak competitors play Producer. The two foraging tactics are incompatible and only individuals playing producer, therefore, can detect prey. I assume that all prey have the same energy value (i.e. *F*) and that only one prey can be discovered and captured at any given time. When a producer captures a prey, it gets the finder's advantage (i.e. a), which corresponds to the quantity of food that it can use exclusively before all the scroungers join and compete for the remaining food. The quantity of food monopolized by each individual then depends on the number of contestants and their respective competitive ability.

Given that strong and weak competitors differ in terms of their capacity to detect prey and their ability to compete for food, their average payoff of producing is then equal to $I_{P}\left(\text{strong}\right)$ and $I_{P}\left(\text{weak}\right)$, respectively, with:

(1) $$I_{P}\left(\text{strong}\right)=\mathrm{\delta a}+\delta \times \left(F-a\right)\times \frac{1}{1+W}$$

and

(2) $$I_{P}\left(\text{weak}\right)=a+\left(F-a\right)\times \frac{\omega }{\omega +W}$$

In both equations, *W* is the sum of the competitive weights of all the scroungers that joined, with:

(3) $$W=\left(G-1\right)\times \left[p\left(1-x\right)+\omega \left(1-p\right)\left(1-y\right)\right]$$

Alternatively, an individual playing Scrounger receives an average payoff equal to $I_{S}\left(\text{strong}\right)$ and $I_{S}\left(\text{weak}\right)$ if it is a strong or a weak competitor, with:

(4) $$I_{S}\left(\text{strong}\right)=\mathrm{pGx}\times \frac{\delta \left(F-a\right)}{2+W_{-1}}+\left(1-p\right)\mathrm{Gy}\times \frac{\left(F-a\right)}{1+\omega +W_{-1}}$$

and

(5) $$I_{S}\left(\text{weak}\right)=\mathrm{pGx}\times \frac{\delta \left(F-a\right)\times \omega }{\varpi +1+W_{-1}}+\left(1-p\right)\mathrm{Gy}\times \frac{\left(F-a\right)\times \omega }{2\omega +W_{-1}}$$

Scroungers join all food discoveries, but the number of discoveries depends on both the number of producers and their phenotype, as the two phenotypes may differ in their efficiency at detecting and capturing prey. Thus, in both equations, the first term corresponds to prey captured by strong competitors, while the second term corresponds to prey captured by weak competitors. Furthermore, the parameter $W_{-1}$ in both equations is the sum of the competitive weights of all the other scroungers that joined (i.e. excluding the focal individual), with:

(6) $$W_{-1}=\left(G-2\right)\times \left[p\left(1-x\right)+\omega \left(1-p\right)\left(1-y\right)\right]$$

### 2.2 Analysis

**FIGURE 1:**
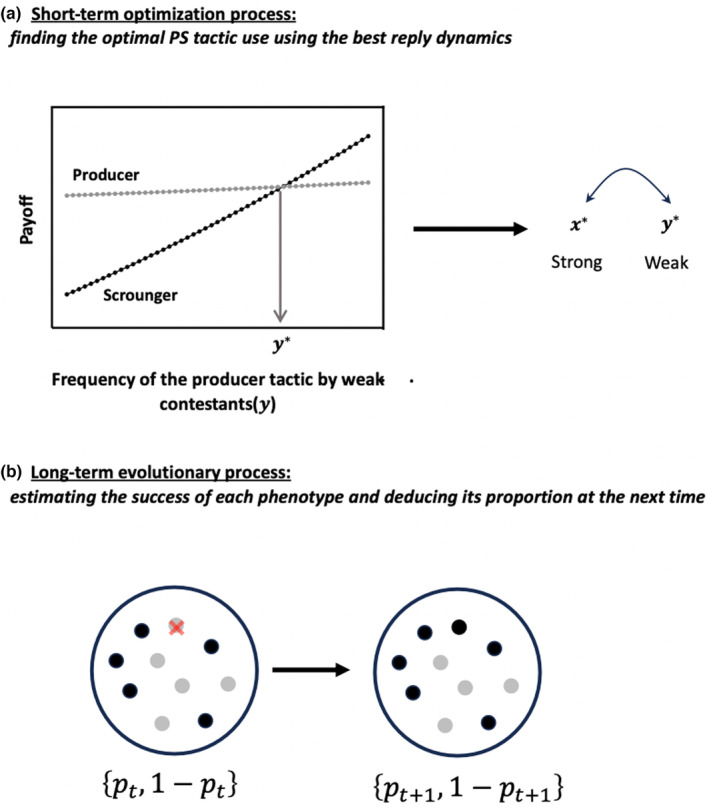
Illustration of the two processes used to (a) predict the optimal producer tactic use of strong (i.e. x*) and weak (i.e. y*) competitors at a given time step, and (b) deduce their average expected gain and relative proportion at the next time step. Panel 1a: Gain of a weak individual who plays producer (grey line) or scrounger (back line) according to the frequency of use of the producer tactic by weak competitors (*y*) when *x* is fixed. In that example, the best response (y*) corresponds to the frequency at which the two payoff lines intersect. The procedure is repeated alternately for strong and weak contestants until finding the Nash equilibrium, that is the values {x*, y*}, such as x* is the best response to y*, and reciprocally. Panel 1b: Knowing the optimal strategy of strong (grey spots) and weak (black spots) contestants, one can calculate their success and deduce their proportion at the next time step.

I varied the ecological and phenotypic parameters of the model (Tables 1 and 2) and, for each set of parameter values, I run the model 1000 consecutive time steps. The model combines a short‐term optimization process and a long‐term evolutionary process: at each time step, I first determine the optimal use of the producer and scrounger tactics using the best reply dynamics (Figure 1a) and then I predict the relative proportion of weak and strong competitors at the next time (Figure 1b) and repeat the same procedure until finding equilibrium values.

#### 2.2.1 Short‐term optimization process

At each time step (i.e. for a fixed value of *p*), I first seek the optimal PS tactic use for weak and strong competitors (i.e. x* and y*) using the best reply dynamics. To do that, I fix the frequency of producer use for strong competitors (i.e. $x_{0}$) and I calculate the expected gain of a weak competitor playing producer or scrounger for any $y_{0}$ ranging from 0 to 1, using Equations (2) and (5) (Figure 1a). I then retain the equilibrium frequency of producer use (i.e. $y_{0}^{*}$), for which all weak competitors obtain the same payoff, so that no player can increase its payoff by changing its tactic use. The stable frequency of producer use can correspond to a pure strategy if one alternative (i.e. producing or scrounging) provides a greater payoff than the other, whatever value $y_{0}$ takes, or a mixed strategy as illustrated in Figure 1a. In that case, the equilibrium frequency of producer corresponds to the frequency at which both payoff curves intersect.

Knowing the optimal producer tactic use for weak competitors (i.e. $y_{0}^{*}$), I calculate the expected gain of a strong competitor playing producer or scrounger for any $x_{1}$ ranging from 0 to 1, using Equations (1) and (4) and, as above, I retain the equilibrium frequency of producer use (i.e. $x_{1}^{*}$), for which all strong competitors obtain the same payoff. Using this solution, I seek again the best response for *y* and so on, until finding the solutions x* and y* of the Nash equilibrium that satisfy: $x_{t}^{*}=x_{t+1}^{*}=x^{*}$ and $y_{t}^{*}=y_{t+1}^{*}=y^{*}$.

From the optimal frequencies of producing for strong and weak competitors, I may estimate an index of specialization γ as:

(7) $$\gamma =\left|x^{*}-y^{*}\right|$$

This index varies between 0, if individuals, irrespective of their phenotype, all behave the same way (e.g. use the two tactics at the same frequencies), and 1 if individuals of the two phenotypes specialize exclusively on one role, each.

#### 2.2.2 Long‐term evolutionary process

Once I have found the optimal frequencies at which strong and weak competitors should play producer $\left\{x^{*},y^{*}\right\}$, I calculate their respective payoffs as:

(8) $$\bar{I}\left(\text{strong}\right)=x^{*}\times I_{P}\left(\text{strong}\right)+\left(1-x^{*}\right)\times I_{S}\left(\text{strong}\right)$$

and

(9) $$\bar{I}\left(\text{weak}\right)=y^{*}\times I_{P}\left(\text{weak}\right)+\left(1-y^{*}\right)\times I_{S}\left(\text{weak}\right)$$

I can then deduce the proportion of strong competitors at the following time step which is equal to:

(10) $$p_{t+1}=\left\{\begin{array}{cc} p_{t} & \text{if}\;\bar{I}\left(\text{strong}\right)=\bar{I}\left(\text{weak}\right)\\ 0.99\times p_{t}+0.01 & \text{if}\;\bar{I}\left(\text{strong}\right)>\bar{I}\left(\text{weak}\right)\\ 0.99\times p_{t}-0.01 & \text{if}\;\bar{I}\left(\text{strong}\right)<\bar{I}\left(\text{weak}\right) \end{array}\right.$$

According to Equation (10), 99% of individuals survive between two consecutive time steps and individuals that die at the end of a given time step are replaced by new individuals of the phenotype with the highest payoff. The same procedure is repeated 1000 consecutive time steps or until we obtain the ESS $\left\{p^{*},x^{*},y^{*}\right\}$ that corresponds to a combination of the equilibrium proportions of strong ($p^{*}$) and weak ($1-p^{*}$) competitors and the frequency at which both phenotypes should adopt the producer tactic, that is x* and y* respectively.

## 3 RESULTS

### 3.1 Short‐term optimization process

**FIGURE 2:**
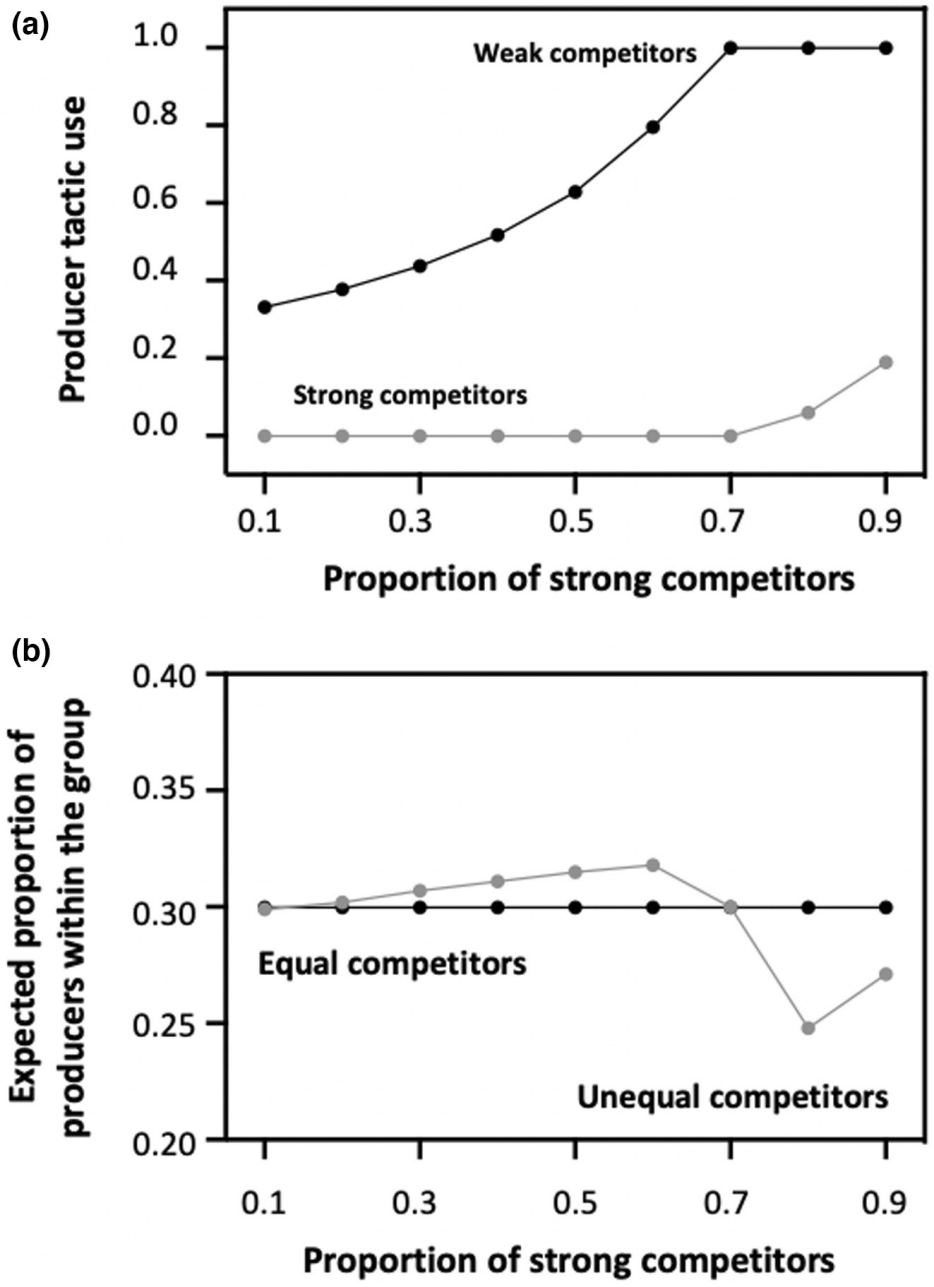
Panel a: Frequency at which strong (grey spots) and weak (black spots) contestants should adopt the Producer tactic in relation to the proportion of strong contestants within the group. Panel b: Resulting expected proportion of producers (grey spots) in relation to the proportion of strong contestants within the group. The predicted values are compared to the values predicted by Vickery et al.'s (1991) model with equal competitors (black spots). In both panels, all parameter values are the default values given in Tables 1 and 2. The depicted values are those predicted at the end of the first time step (assuming that the group composition remains stable).

**FIGURE 3:**
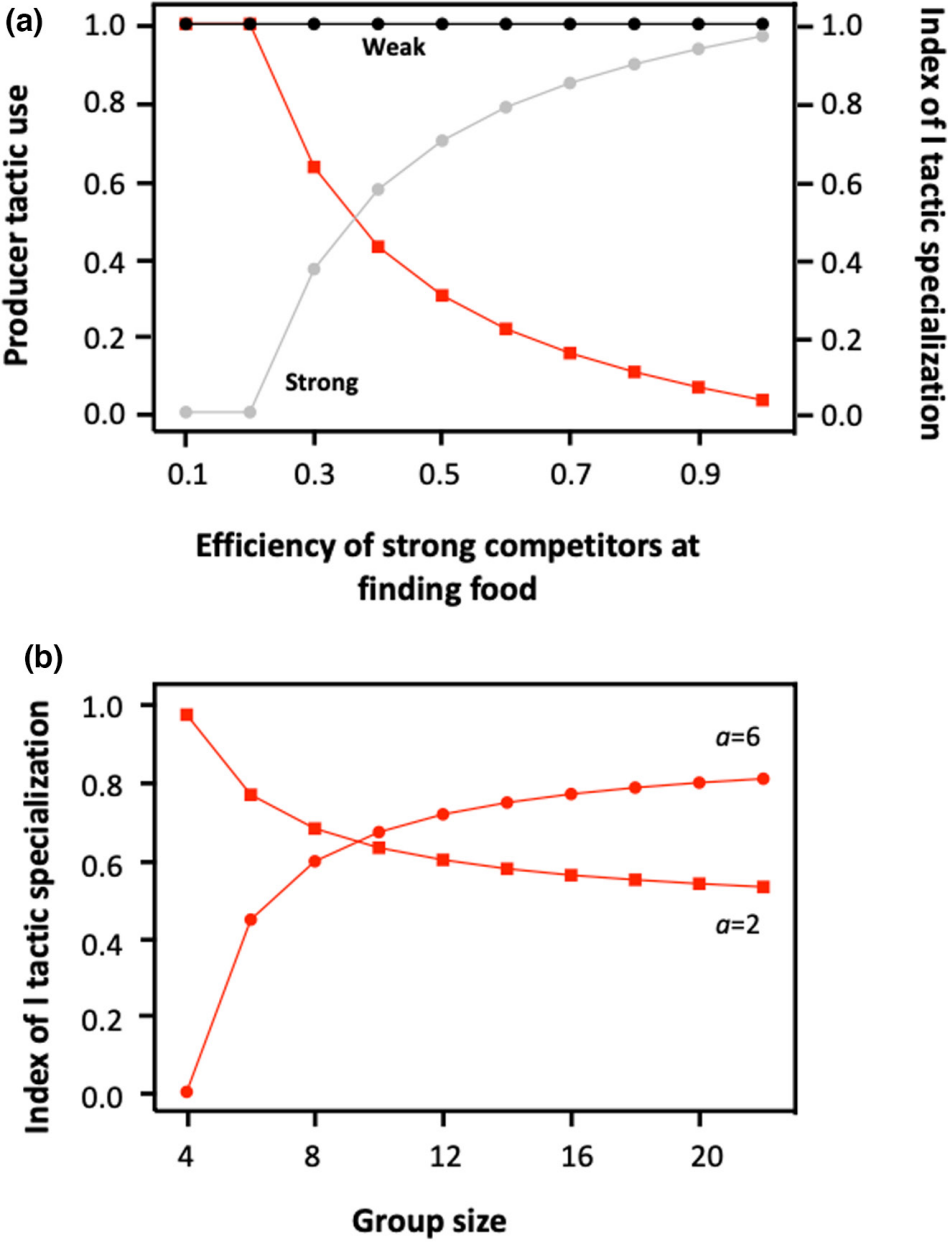
Frequency at which strong (grey spots) and weak (black spots) contestants should adopt the Producer tactic and degree of tactic specialization (red spots) in relation to the capacity of strong competitors to detect and capture prey (*δ*). The depicted values are those predicted at the end of the first time step (assuming that the group composition remains stable) with all parameter values being the default values given in Tables 1 and 2, except a=8 in Panel a.

When the composition of the group is stable, the model predicts that the mean expected frequency of producers and scroungers depends on the relative proportion of strong and weak contestants. Logically, strong competitors always rely more on scrounging compared to weak competitors, but the frequency at which individuals of both phenotypes should produce and scrounge, and hence the degree of tactic specialization, varies with their relative proportion (Figure 2a). Specifically, in groups with very few strong competitors, the model predicts that strong competitors should specialize on scrounging, while weak competitors should alternate between producing and scrounging. However, given that their success as scroungers decreases as the proportion of strong competitors increases, they should rely less and less on scrounging as they become increasingly rare, until they specialize on producing. From that point, an increase in the proportion of strong competitors leads to a decrease in the number of prey captured by weak competitors, which may force stronger competitors to rely more and more on the producer tactic. Thus, all other parameters being equal, the model predicts that differences among groups in their composition (i.e. in the proportion of strong vs. weak contestants) may be associated with differences in the mean PS tactic use (Figure 2b) as well as in the level of foraging tactic specialization. Reliance on the producer tactic by strong competitors is also affected by their efficiency at finding food, meaning that the degree of foraging tactic specialization should depend on the type of resources exploited, and more particularly on whether they require particular skills that trade‐off with competitiveness. Strong competitors are indeed expected to specialize exclusively on the scrounger role when they are highly ineffective at finding food, while they should rely more and more on the producer tactic, hence leading to a decrease in tactic specialization, as they become increasingly efficient at finding food by themselves (Figure 3a). Note, however, that an increase in producer tactic use by strong competitors is expected only when the benefits of producing are strong, and hence when the finder's advantage is large. By contrast, in conditions which favour scrounging (i.e. low values of *a*), strong contestant should always specialize on the scrounger role even when they are as effective as weak contestants in detecting and capturing prey. The degree of tactic specialization is also influenced by group size, though increasing competitor number may cause either a decrease or an increase in the degree of tactic specialization, depending on the characteristics of the resources exploited, and particularly, again, on the finder's advantage (Figure 3b). Specifically, in conditions that promote producing (i.e. when the finder's advantage is large), individuals, irrespective of their phenotype, mainly (or exclusively) rely on the producer tactic when foraging in small groups, where there is thus little tactic specialization. Increasing group size then leads to an increase in the use of scrounger tactic by strong competitors and hence in the degree of tactic specialization. By contrast, in conditions that favour scrounging (i.e. when the finder's advantage is small), the degree of tactic specialization should be lower within large groups. This arises because each phenotype specializes exclusively on one role (i.e. weak competitors specialize on producing while strong competitors specialize on scrounging) when group size is small, while weak competitors rely more and more on the scrounger tactic in larger groups.

### 3.2 Long‐term evolutionary process

**FIGURE 4:**
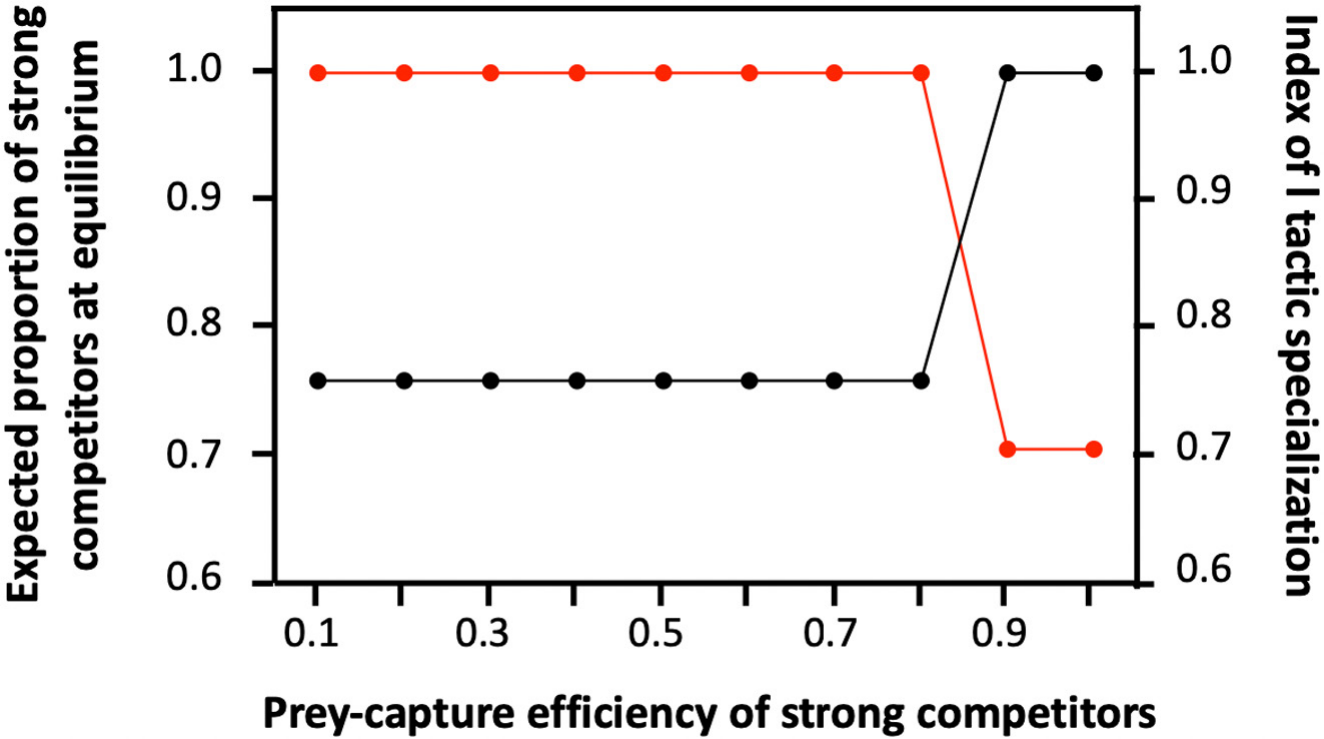
Equilibrium proportion of strong contestants (black spots) and expected degree of individual specialization (red spots) after 1000 consecutive time steps in relation to the ability of strong competitors to detect and capture prey. All parameter values are the default values given in Tables 1 and 2, except w=0.5.

**FIGURE 5:**
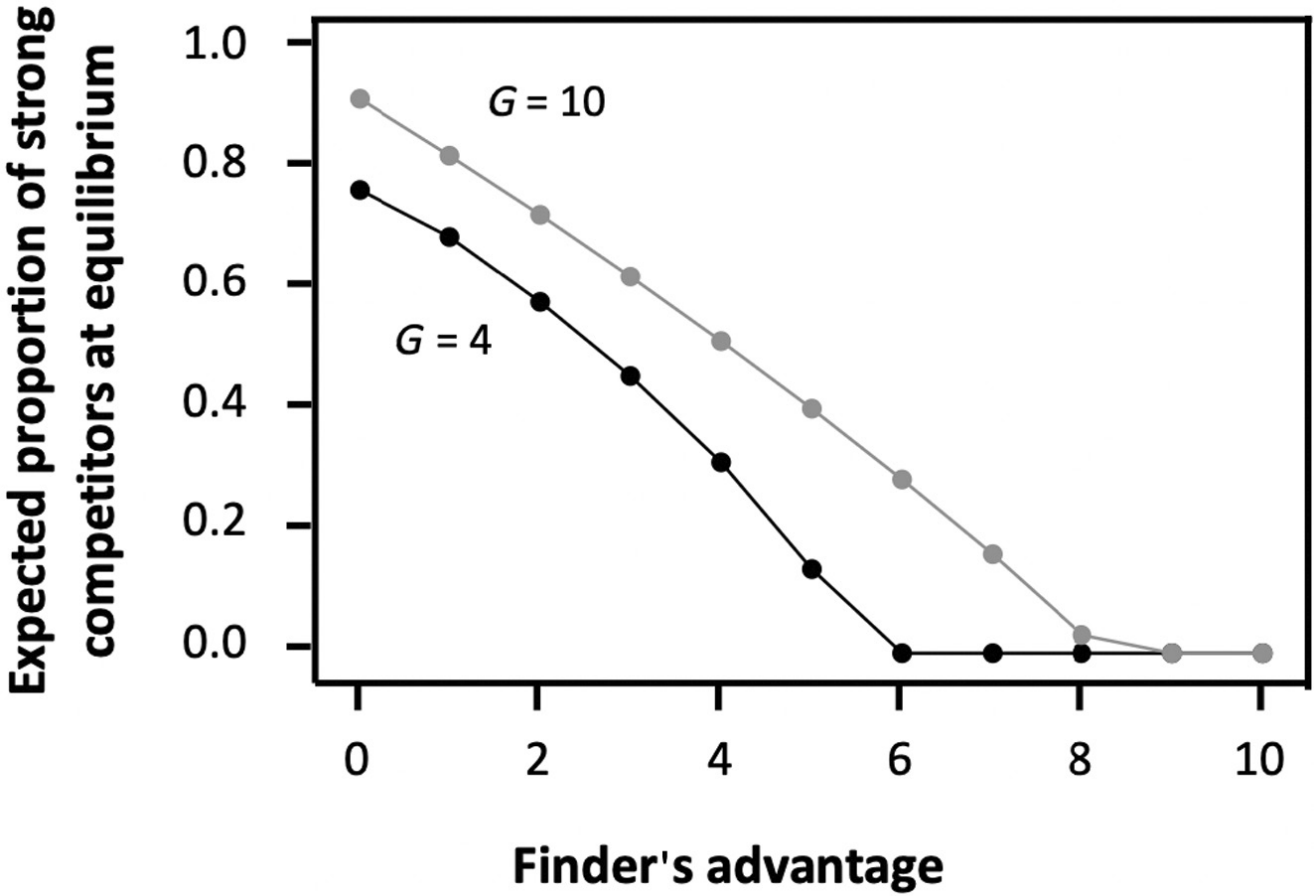
Equilibrium proportion of strong contestants (grey spots) after 1000 consecutive time steps in relation to the finder's advantage within two different group sizes. All parameter values are the default values given in Tables 1 and 2.

Despite all individuals being capable of adjusting their PS tactic use to maximize their payoff at each time step, strong and weak contestants may experience different payoffs, leading to a change in their relative proportion over time. In particular, the model predicts that natural selection can lead to a loss of phenotypic diversity under certain conditions. This logically happens when the two phenotypes only differ regarding one of the two abilities (i.e. competitive ability or prey‐capture ability), and hence when there is no trade‐off between the two. For instance, when strong contestants are as effective as weak contestants in detecting and capturing prey, but are more efficient in monopolizing access to resources, natural selection should eliminate weak contestants over time (Figure 4). Strong individuals are then forced to use both foraging tactics. Inversely, when weak contestants are as effective as weak contestants in monopolizing access to resources, but are more efficient in detecting and capturing prey, natural selection should eliminate strong contestants over time, forcing weak contestants to use both foraging tactics (Figure S1). By contrast, when the ability to detect prey trades‐off with competitive ability (and hence when better competitors are less efficient at detecting and capturing prey), the two phenotypes coexist for most parameter values and each phenotype specializes exclusively on one role (i.e. weak competitors specialize on producing while strong competitors specialize on scrounging, Figure 4 and Figure S1). Yet, the relative proportions of strong and weak contestants vary depending on environmental conditions. Specifically, as strong competitors specialize on scrounging, the stable proportion of strong competitors at equilibrium (i.e. once the group has reached the ESS) is higher in conditions promoting scrounging. For that reason, the model predicts that the proportion of strong contestants, at equilibrium, should be higher when the finder's advantage is low as well as in large groups (Figure 5) as these conditions favour individuals that are more efficient at aggressively defending prey.

## 4 DISCUSSION

Consistent differences among individuals in their diet, niche use and/or foraging tactic use are widespread within social groups (e.g. Araujo et al., 2011; Montiglio et al., 2013), but the mechanisms explaining the evolution and maintenance of individual specialization are still little understood. Using a PS game, here I demonstrate that individuals may specialize on one or the other foraging tactic if their success as a producer or as a scrounger depends on their phenotype. The existence of individual differences in competitive abilities may thus be sufficient to explain short‐term individual foraging tactic specialization. Yet, the degree of individual specialization within a group is influenced by both the social and ecological context. Furthermore, the maintenance of consistent phenotypic differences, that are necessary for stable tactic specialization, require the existence of a trade‐off between abilities that enable greater success as either scroungers or producers.

The model predicts that strong and weak competitors should rely more on scrounging and producing, respectively, which is consistent with previous empirical and theoretical findings (Bicca‐Marques & Garber, 2005; Jones et al., 2017; Lendvai et al., 2006; Liker & Barta, 2002; Philips et al., 2018). Yet, the degree of tactic specialization is predicted to vary according to ecological and social factors, including the size and composition of the group or the prey value. For instance, reliance of the producer tactic by strong competitors should expectedly increase as the proportion of strong competitors within the group increases. For that reason, even if an individual tends to specialize on a particular foraging tactic within a foraging group whose composition is stable, it might use both tactics at different frequencies within another group, all other factors being equal. Changes in the group composition (i.e. relative proportion of strong and weak competitors) may thus also lead to changes in the average PS tactic use. This prediction is supported by the results of Morand‐Ferron et al.'s (2011) experiment, in which birds exhibited significant individual differences in tactic use that persisted over time but only when the social environment (i.e. composition of the group) was constant. The model also predicts that increasing competition (i.e. the number of competitors) should lead to increased or decreased tactic specialization.

It is well recognized that competition has a profound impact on individual specialization (Sheppard et al., 2018). In particular, competition theory predicts that increased competition should favour reduced dietary overlap as it would allow individuals to reduce conflicts, and, as such, should increase individual specialization (Pianka, 1981; Schoener, 1974). Supporting the competition theory, results from the present model demonstrate that increased competition, under conditions that favour a high level of scrounging, promotes tactic specialization, though for a different reason. Indeed, the competitive theory relies on the assumptions that individuals, although in groups, only acquire their resources individually (i.e. through their own efforts) and do benefit from avoiding aggressive interactions. However, despite the potential costs of aggressive interactions, scrounging, almost systematically, provides higher payoffs than producing when scroungers are rare, and hence would be an unavoidable behaviour. Because the expected frequency of scrounging events increases with increasing group size, this should then cause the strongest contestants to specialize more and more on scrounging, thereby increasing foraging tactic specialization.

Results from the present study are consistent with previous studies reporting that frequency dependence might promote behavioural specialization, when individuals engaged in games adjust, through learning, their behaviour according to ecological conditions and social context to maximize their fitness (Dall et al., 2004; Dubois et al., 2012; Leimar et al., 2022). Yet, if there are inherent differences among individuals that determine their producing success or ability to scrounge, one phenotype may accrue higher payoffs than the other one (Barta & Giraldeau, 1998; Liker & Barta, 2002). Assuming that the foraging success of an individual is related to its ability to survive and/or reproduce, such differences in payoffs should then cause a change in the proportion of both phenotypes over time, until the worst performing phenotype is eliminated, or the two phenotypes reach an equilibrium where they are both maintained in the group and achieve equal fitness. The present study demonstrates that this latter situation only occurs when strong competitors are more efficient than weak competitors in stealing food, but weak competitors are more efficient than strong competitors in discovering and capturing prey. Life‐history trade‐offs, as the competition‐colonization trade‐off (Levins & Culver, 1971), have been proposed to explain species coexistence. According to this mechanism, two species can indeed theoretically coexist if the species with an inferior competitive ability is better at colonizing new habitats. As demonstrated here, this concept can thus be expanded to intraspecific competition as well as traits that affect the same fitness component (e.g. foraging success). When prey can be stolen, one might then expect individual differences in cognitive skills to persist over time even in harsh environmental conditions (in which prey are scarce) that should a priori favour enhanced spatial memory (Lee & Thornton, 2021). Supporting this conclusion, I found that the maintenance of strong competitors was conditional to the possibility to scrounge: strong competitors logically were eliminated over time when the finder's advantage was high (and hence when the amount of resources that could be stolen was negligible). The opportunity to scrounge is then an essential aspect to consider when aiming at predicting the role of ecological conditions on the evolution of cognition if prey capture is cognitively demanding. For instance, although urbanization might cause a decline in cognitive performances (mainly because food is more abundant in urbanized environments and individuals, therefore, need to rely less on cached food), there is no clear evidence for a negative effect of urbanization on spatial memory performance (Lee & Thornton, 2021; Thompson & Morand‐Ferron, 2019; Vincze & Kovacs, 2022). This could be explained by the fact that even if individuals rely more on cached foods in non‐urban habitats, the opportunity to steal others' caches may exert a disruptive effect and as such, increase the variation among individuals in their cognitive and competitive abilities, but with no impact on the average traits. The present model assumes that the contestants' ability to find or steal food is fixed over time, and, as such, does not provide predictions to directly support this hypothesis. However, it predicts that contestants with high competitive ability may be maintained within populations, despite their inefficiency at detecting prey, which strongly suggests that the possibility of scrounging might affect the magnitude of the trade‐off between these two abilities.

## 5 CONCLUSIONS

Results from the present study confirm the expectation that the maintenance of persistent individual differences requires a trade‐off between different abilities that enable greater success as either a producer or a scrounger. Furthermore, if the PS game has been used as a general framework for addressing this question, its conclusions may be generalized to other games and other individual trade‐offs. Overall, its findings highlight the importance of considering the existence of alternative tactics (such as pilferage) to measure and predict the evolution of traits, such as cognitive traits, within populations (Dubois et al., 2010; Reichert et al., 2021; Vernouillet, 2021) and thus predict their potential to cope with environmental change. Finally, though it is based on several simplifying assumptions, the model generates several testable predictions that can be useful to improve our understanding of the mechanisms underlying individual specialization within social groups.

## AUTHOR CONTRIBUTIONS

**Frédérique Dubois:** Conceptualization (equal); formal analysis (equal); funding acquisition (equal); methodology (equal); writing – original draft (equal).

## CONFLICT OF INTEREST STATEMENT

The author declares no conflict of interests.

## Supporting information


Data S1

Figure S1

## Data Availability

The simulation code is available as supplementary material (Data S1).
