# Supplementary material for: Skill trade‐offs promote persistent individual differences and specialized tactics
Source: Ecol Evol. 2023 Oct 4;13(10):e10578. doi: 10.1002/ece3.10578 (PMC10550786; doi:10.1002/ece3.10578)
Supplement: Supplementary file 1 — Data S1 [file ECE3-13-e10578-s002.docx]

#include<stdio.h>

#include<stdlib.h>

#include<math.h>

#include<time.h>

FILE*file_simul;

void main (void){

double p=0.5; /*Initial proportion of strong competitors*/

int a=6; /*finder's advantage*/

int F=10; /*Prey value*/

int G=22; /*Group size*/

double d=0.8; /*Efficiency of strong competitor to detect prey*/

double w=0.8; /*Competitive weight of weak competitors*/

int i,t;

double x,y; /*Frequency at which strong and weak competitors play Producer*/

double IP_strong,IS_strong,IP_weak,IS_weak; /*Payoffs expected by strong and weak competitors if they play Producer or Scrounger*/

double Payoff_strong,Payoff_weak; /*Expected gain of a strong and weak competitor*/

double prop,Dif_Payoff,dif;

double sol,opt_x,opt_y;

int negatif,positif;

double Wp; /*Sum of the competitive weights of all scroungers when the focal individual plays producer*/

double Ws; /*Sum of the competitive weights of all other scroungers excluding the focal individual that plays scrounger*/

for (t=0;t<1000;t++){

x=0.5;

printf("\nTime %d",t);

**/**Finding the optimal PS tactic use using the best reply dynamics**/**

for(i=0;i<50;i++){

negatif=0;

positif=0;

dif=10;

for (y=0;y<=1;y+=0.0001){

Wp=(G-1)*(p*(1-x)+w*(1-p)*(1-y));

Ws=(G-2)*(p*(1-x)+w*(1-p)*(1-y));

IP_weak=a+((F-a)*w)/(w+Wp);

IS_weak=(p*G*x*d*(F-a)*w)/(w+1+Ws)+((1-p)*G*y*(F-a)*w)/(2*w+Ws);

Payoff_weak=y*IP_weak+(1-y)*IS_weak;

if((IP_weak-IS_weak)>0)positif+=1;

if((IP_weak-IS_weak)<0)negatif+=1;

if(abs(IP_weak-IS_weak)<dif){

sol=y;

dif=abs(IP_weak-IS_weak);

}

}

if (negatif==0)opt_y=1;

else if (positif==0)opt_y=0;

else opt_y=sol;

/*printf("\nOptimal value of y:%lf",opt_y);*/

y=opt_y;

dif=10;

negatif=0;

positif=0;

for (x=0;x<1;x+=0.0001){

Wp=(G-1)*(p*(1-x)+w*(1-p)*(1-y));

Ws=(G-2)*(p*(1-x)+w*(1-p)*(1-y));

IP_strong=d*a+(d*(F-a))/(1+Wp);

IS_strong=(p*G*x*d*(F-a))/(2+Ws)+((1-p)*G*y*(F-a))/(1+w+Ws);

Payoff_strong=x*IP_strong+(1-x)*IS_strong;

if((IP_strong-IS_strong)>0)positif+=1;

if((IP_strong-IS_strong)<0)negatif+=1;

if(abs(IP_strong-IS_strong)<dif){

sol=x;

dif=abs(IP_strong-IS_strong);

}

}

if (negatif==0)opt_x=1;

else if (positif==0)opt_x=0;

else opt_x=sol;

/*printf("\nOptimal value of x:%lf",opt_x);*/

x=opt_x;

}

printf("\nOptimal producer tactic use for strong competitors:%lf",x);

printf("\nOptimal producer tactic use for weak competitors:%lf",y);

/* ***Estimating the success of each phenotype and deducing its proportion at the next time****/

Wp=(G-1)*(p*(1-x)+w*(1-p)*(1-y));

Ws=(G-2)*(p*(1-x)+w*(1-p)*(1-y));

IP_weak=a+(F-a)*w/(w+Wp);

IS_weak=p*G*x*d*(F-a)*w/(w+1+Ws)+(1-p)*G*y*(F-a)*w/(2*w+Ws);

IP_strong=d*a+d*(F-a)/(1+Wp);

IS_strong=(p*G*x*d*(F-a))/(2+Ws)+(1-p)*G*y*(F-a)/(1+w+Ws);

Payoff_strong=x*IP_strong+(1-x)*IS_strong;

Payoff_weak=y*IP_weak+(1-y)*IS_weak;

Dif_Payoff=abs(Payoff_strong-Payoff_weak);

printf("\nPayoff Strong:%lf Weak:%lf",Payoff_strong,Payoff_weak);

if (Dif_Payoff<0.001)prop=p;

else if ((Dif_Payoff>=0.001)&&(Payoff_strong>Payoff_weak)) prop=p*0.99+0.01;

else prop=p*0.99-0.01;

if (prop>1)prop=1;

if (prop<0) prop=0;

printf("\nProportion of strong competitors:%lf\n",prop);

p=prop;

}

}
