# Supplementary figures and images for: Skill trade‐offs promote persistent individual differences and specialized tactics
Source: Ecol Evol. 2023 Oct 4;13(10):e10578. doi: 10.1002/ece3.10578 (PMC10550786; doi:10.1002/ece3.10578)

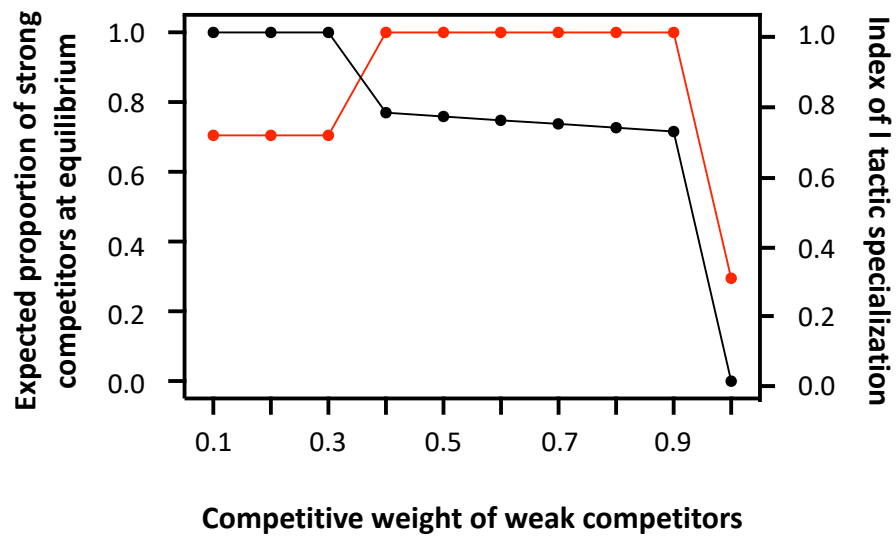

Supplement: Supplementary file 2 — Figure S1 [file ECE3-13-e10578-s001.pdf]
